# The Development of a Magnesium-Releasing and Long-Term Mechanically Stable Calcium Phosphate Bone Cement Possessing Osteogenic and Immunomodulation Effects for Promoting Bone Fracture Regeneration

**DOI:** 10.3389/fbioe.2021.803723

**Published:** 2022-01-11

**Authors:** Jun Wu, Feihong Liu, Zejin Wang, Yuan Liu, Xiaoli Zhao, Christian Fang, Frankie Leung, Kelvin W. K. Yeung, Tak Man Wong

**Affiliations:** ^1^ Shenzhen Key Laboratory for Innovative Technology in Orthopaedic Trauma, The University of Hong Kong-Shenzhen Hospital, Shenzhen, China; ^2^ Department of Orthopaedics and Traumatology, The University of Hong Kong, Pokfulam, Hong Kong SAR, China; ^3^ Research Center for Human Tissues and Organs Degeneration, Institute of Biomedicine and Biotechnology, Shenzhen Institutes of Advanced Technology, Chinese Academy of Sciences, Shenzhen, China

**Keywords:** calcium phosphate cement (CPC), immunomodulation, osteogenic, bone regeneration, anti-collapsibility

## Abstract

Bone grafts are commonly used for the treatment of critical sized bone defects. Since the supply of autologous bone is insufficient, allogeneic bone grafts have been used most of the time. However, the poor osteogenic property of allogeneic bone grafts after pretreatment results in delayed union, non-union, or even occasional deformity. Calcium phosphate cement (CPC) is one of the most promising bone filling materials due to its good biocompatibility and similar chemical components as natural bone. However, clinical applications of CPC were hampered by limited osteogenic effects, undesired immune response which results in resorption, and poor mechanical stability *in vivo*. Magnesium (Mg) has been proven to trigger bone regeneration through modulating cell behaviors of mesenchymal stem cells and macrophages significantly. Unfortunately, the degradation raters of pure Mg and Mg oxide are extremely fast, resulting in early collapse of Mg contained CPC. In this study, we developed a novel magnesium contained calcium phosphate bone cement (Mg-CPC), possessing long-term mechanical stability and osteogenic effects through sustained release of Mg. Furthermore*, in vitro* studies showed that Mg-CPC had no cytotoxic effects on hBMMSCs and macrophage RAW 264.7, and could enhance the osteogenic differentiation as determined by alkaline phosphate (ALP) activity and calcium nodule staining, as well as suppress the inflammatory as determined by expression of anti-inflammatory cytokine IL-1RA. We also found that Mg-CPC promoted new bone formation and bone maturation *in vivo*. These results suggest that Mg-CPC should be a good substitute material for bone grafts in clinical use.

## 1 Introduction

Each year, a large number of people suffer from critical sized bone defects caused by high-energy trauma or diseases (Wiese and Pape, 2010). The repair of critical bone defect has always been a clinical challenge (Zhao et al., 2009). Autologous bone grafting is the preferred treatment option in many cases of orthopedic surgeries (Bauer and Muschler, 2000; Jung et al., 2020). But for critical sized bone defects, the donor sites for autologous bone harvest are always insufficient, and a second operation is sometimes needed, leading to additional pain and increased complication risks (Arrington et al., 1996). Allogeneic bone graft is another choice (De Long et al., 2007). However, to eliminate immune rejection and infection, pretreatment inactivation is needed before implantation, which decreases the osteogenic activity as well (Zimmermann and Moghaddam, 2011), resulting in delayed healing, non-union, or even occasional deformities (Orchard et al., 2014). Therefore, scientists have long attempted to develop novel implants with good biocompatibility and osteogenic effects (Reichert et al., 2009).

Calcium phosphate bone cement (CPC) is a biological material with good biocompatibility, bone conductivity, and similar mechanical strength to cancellous bone. The final product formed after curing is hydroxyapatite (HA), which is similar to the main inorganic component of natural bone (Nair et al., 2013) and is considered one of the most promising potential bone graft substitutes (Scheer and Adolfsson, 2009). CPC was invented by Brown and Chow in the 1980s (Brown and Chow, 1983) and was approved for clinical use by the FDA in 1996 (Friedman et al., 1998). However, CPC has limited osteogenic effects, and implant failures due to delayed healing or non-union have been observed. Many researchers have tried to add osteogenic substances, such as growth factors, stem cells and so on, into bone cement to improve the osteogenesis of bone cement (Meraw et al., 2000; Zhao et al., 2011), but few have explored the effect of immune response, which plays important roles in modulating bone regeneration after a fracture. Moreover, prolonged inflammation is one of the major causes of implant failure (Han et al., 2014; Kovach et al., 2015).

Biomaterial implants inevitably cause immune response to the host and have a profound impact on the process of bone healing (Franz et al., 2011). Macrophages have long been considered to be important immune-benefit cells, and their polarization can be divided into two types: classical activation into inflammatory macrophages M1-type and selective activation into therapeutic macrophages M2-type (Stein et al., 1992). M1-type macrophages can produce pro-inflammatory cytokines, which promote inflammation and affect wound healing. M2-type macrophages can produce anti-inflammatory cytokines, which stimulate arginase activity and promote wound healing. They can be converted into each other under certain conditions (Mosser and Edwards, 2008).

Recent studies have reported the application of magnesium implants in fracture healing. Chen et al. (2014) prepared a kind of β-TCP scaffold containing an Mg coating that could effectively induce the differentiation of macrophages into M2 compared with uncoated β-TCP, indicating that magnesium has the potential of immune regulation of the bone. Wang et al. (2016) prepared a magnesium-containing CPC by mixing the cement derived from magnesium oxide (MgO) in a fixed ratio on the basis of ordinary CPC. *In vitro* experiments showed that this bone cement could not only promote osteogenesis but also effectively reduce pro-inflammatory cytokines. However, the underlining mechanisms of modulating bone immunology by magnesium-containing CPC are still to be revealed.

Therefore, it is of great interest to incorporate magnesium into orthopedic implants to increase osteogenic effects and to modulate immune responses for bone regeneration. However, magnesium-based implants usually degrade too fast under certain physiological conditions, which causes the early collapse of CPC and hampers their clinical applications. In the previous study, our research group synthesized a strontium-containing CPC possessing bone regeneration-promoting effects that could rapidly self-solidify at room temperature. In this study, a novel magnesium-containing calcium phosphate bone cement (Mg-CPC) was developed by incorporating a magnesium compound with a compatible degradation rate, combined with an organic cross-linking agent to achieve collapse resistance, sustained magnesium release, and long-term mechanical stability. To reveal the mechanism of enhanced bone healing by Mg, the osteogenic and anti-inflammatory properties of the Mg-CPC and CPC were tested *in vitro* and *in vivo*. The results showed that the Mg-CPC could enhance osteogenic differentiation and suppress prolonged inflammation. Furthermore, the sustained release of magnesium contributed to the in-growth of new bone tissue, which facilitated the union of Mg-CPC and bone tissue, while the mechanical strength of Mg-CPC was not deteriorated. The presented Mg-CPC might be used in promising applications in healing critical sized bone defects.

## 2 Materials and Methods

### 2.1 Fabrication of the Magnesium-Releasing Calcium Phosphate Bone Cement

The calcium phosphate cement was synthesized by thoroughly stirring the liquid phase and the powder phase (Table 1). The liquid phase consists of 20% (wt%) citric acid (sigma) and 12% (wt%) polyvinylpyrrolidone K-30 (PVP, sigma) in ultrapure water. The powder phase consists of tetracalcium phosphate (TTCP, Wako) and dicalcium phosphate anhydrous (DCPA, sigma). We used magnesium phosphate dibasic trihydrate (DMPA, sigma) to make Mg-CPC. The cement paste was prepared by mixing the liquid and powder phases at a ratio of 0.7 ml/g. In this study, the power and liquid phases of cement with different magnesium contents (0, 5, 10, and 20%) were fully mixed and injected into the customized mold to form cylindrical samples with a bottom diameter of 6 mm and a height of 12 mm and then removed after curing and ethylene oxide sterilization. The curing reaction of classical calcium phosphate cement can be divided into two stages: hydration and precipitation. The phosphate compound of calcium firstly produces a large number of calcium ions and phosphate ions through hydration, which then react slowly to form HA (Eqs 1, 2, 6; Liu et al., 2003).

**TABLE 1:** The powder composition of calcium phosphate bone cement.

Group	Mg/(Mg + Ca) (mole ratio)	TTCP [Ca_4_(PO_4_)_2_O] (mole ratio)	DCPA [CaHPO_4_] (mole ratio)	DMPA [MgHPO_4_] (mole ratio)
CPC	0	1	1	0
5% Mg-CPC	5%	1	0.75	0.25
10% Mg-CPC	10%	1	0.5	0.5
20% Mg-CPC	20%	1	0	1

The chemical reaction equation is as follows

(a) Dissolution

$$Ca_{4}\left(PO_{4}\right)+H_{2}O\to 4Ca^{2+}+2PO_{4}^{3-}+2OH^{-}$$ (1)

$$CaH\left(PO_{4}\right)\to Ca^{2+}+2HPO_{4}^{2-}$$ (2)

$$MgH\left(PO_{4}\right)\to Mg^{2+}+2HPO_{4}^{2-}$$ (3)

(b) Chelation

$$3Mg^{2+}+2H_{3}Cit\to Mg_{3}Cit_{2}$$ (4)

$$3Ca^{2+}+2H_{3}Cit\to Ca_{3}Cit_{2}$$ (5)

(c) HA formation

$$10Ca^{2+}+6PO_{4}^{3-}+2OH^{-}\to Ca_{10}{\left(PO_{4}\right)}_{6}{\left(OH\right)}_{2}$$ (6)

### 2.2 Material Characterizations

#### 2.2.1 Surface Morphology and Chemical Composition

The cement samples were immersed in simulated body fluid (SBF) solution and placed in a 37°C incubator. After immersion for 0, 14, and 28 days, quenching with liquid nitrogen for 30 min to stop the setting reaction of cements. Then the cements were dried using a freeze-drying machine (Alp2-4LD, Christ, Germany) for 15 h. The surface structure of the bone cement was observed using a scanning electron microscope (SEM) (ZEISS SUPRA R 55, Zeiss, Germany), and the surface distribution of calcium and magnesium was determined by energy dispersive spectrometer (EDS) after being gold plated. Each cement was ground into a powder using an amber mortar and analyzed by X-ray diffraction (XRD) (D8 Advance, Bruker, Germany) using Cu Kα (k = 1.5406 Å) radiation in step-scan mode (2y = 0.02 per step). Three samples were tested in each group.

#### 2.2.2 Compressive Strength and Setting Time

The CPC and Mg-CPCs were made into a cylinder with a diameter of 6 mm and a height of 12 mm by using a mold. After setting and incubate at 37°C for 24 h. The cement samples were immersed in SBF for 0 and 28 days at 37°C. The volume (ml) of SBF was determined according to the equation of V = S/10, in which the S is the surface area of the cements (mm^2^). The SBF was changed every 3 days. The material test machine (Instron E10000, United States) was taken out under a load of 1KN, and the speed was 0.1 mm/min, until the cement breaks. Three samples were tested in each group.

The setting time of cements were tested using the Gillmore apparatus according to the ASTM: C266-89 standard. Which have two kinds of needles, the light and thick needle with a 113.4 g weight and 2.13 mm diameter needle tip, the heavy and thin needle with 453.6 g weight and 1.06 mm diameter needle tip. After fully mixed the liquid and powder phases, moved into the customized mold with a bottom diameter of 6 mm and a height of 12 mm, two needles with different diameters and weights were gently placed on the cement surface. The initial setting time was measured by a light and thick needle. When the cement surface has no visual marks of the needle tip, record the time. Each test was repeated three times.

#### 2.2.3 *In Vitro* Ion Release

The CPC and Mg-CPCs were made into a cylinder with a diameter of 4.5 mm and a height of 6 mm by using a mold. After setting and incubate at 37°C for 24 h, the cement samples were immersed in an 8 ml phosphate buffer saline (PBS) solution (without calcium and magnesium ions, PH = 7.35) and placed in a 37°C incubator. At days 1, 3, 7, 14 and 28 after immersion, all the extracts were collected and replaced with fresh PBS. An inductively coupled plasma emission spectrometer (Perkin Elmer, Optima 7000, United States) was used to detect the concentration of Mg and Ca ions in the extracted samples. The process was repeated three times for each group of samples.

### 2.3. *In Vitro* Characterizations

#### 2.3.1. Cell Culture

The murine-derived macrophage cell line RAW 264.7 cells (RAW cells, Cell Bank, purchased from the Chinese Academy of Sciences) and human bone marrow mesenchymal stem cells (hBMMSCs) were purchased from Cyagen Biosciences Inc. (Guangzhou, China). The RAW 264.7 cells were cultured using the Dulbecco’s modified Eagle’s medium (DMEM, Gibco) supplemented with 10% (v/v) fetal bovine serum (FBS, Gibco) and antibiotics (100 U/ml of penicillin and 100 mg/ml of streptomycin) (Thermo Fisher Scientific, United States). The hBMMSCs were cultured using Minimal Essential Medium Alpha (α-MEM, Gibco) supplemented with 10% (v/v) FBS and antibiotics (100 U/ml of penicillin and 100 mg/ml of streptomycin).

#### 2.3.2 Cell Viability of Human Bone Marrow Mesenchymal Stem Cells and RAW264.7

The hBMMSCs were seeded on the 96-well plate with a density of 0.5 × 10^4^ in each well at 37°C with 5% CO_2_ for 24 h; then the medium was removed, and the extract was added to each well. At days 1 and 3, cell viability was tested using Cell Counting Kit-8 (Dojindo, Japan). The RAW264.7 macrophages at a density of 10,000 cells per well in the 96-well plate were incubated at 37°C and supplemented with 5% CO_2_ for 1 d and then cultured with the extraction solution. The optical density (OD) was tested by a microplate reader (Epoch, BioTek, United States) at an absorbance of 450 nm. The extract solution was prepared according to ISO 10993-5, and the cements were immersed in the culture medium for 24 h at a ratio of 0.2 g/ml.

#### 2.3.3 Alkaline Phosphatase Activity and ECM Mineralization of Human Bone Marrow Mesenchymal Stem Cells

The hBMMSCs were seeded on a 48-well plate (with a density of 2 × 10^4^ for each well), incubated at 37°C, and supplemented with 5% CO_2_. The cells were cultured using α-MEM supplemented with 10% (v/v) FBS and antibiotics. After 24 h, the culture mediums were replaced with extracts of CPC or Mg-CPC; for the Alkaline phosphatase activity (ALP) activity test, osteogenic differentiation materials [10 mM β-glycerophosphate (sigma), 50 μM ascorbic acid (sigma), and 10 nM dexamethasone (sigma)] were added to the mediums. After incubation for 3, 7, and 14 days, the cells were washed three times using PBS and then lysed by 0.1% Triton X-100 at 4°C for 30 min. The ALP activity was determined using an alkaline phosphatase assay kit (Nanjingjianchen, China). After incubation for 21 days, the cells were washed three times using PBS, fixed on ice with paraformaldehyde for 30 min, and washed again three times with PBS.

#### 2.3.4 Immune Modulation Effects of Magnesium Contained Calcium Phosphate Bone Cement

##### 2.3.4.1 Immunofluorescence

For the primary antibodies and antibodies, iNOS (Abcam, United States) and arginase (Abcam, United States) were chosen as markers for M1 and M2, respectively. RAW 264.7 cells were inoculated on the cement samples and cultured for 24 h. The supernatant was abandoned, and the cells on CPCs were fixed by 4% paraformaldehyde for 30 min. After fixation, the samples were washed three times using PBS and permeabilized with 0.25% Triton X-100 (PBST) for 10 min. They were then washed three times again using PBS and blocked for 30 min at room temperature using 5% FBS. The cells were then incubated with primary antibodies at 4°C overnight, washed again three times with PBS, and incubated with secondary antibodies (Abcam, United States) for 1 h at room temperature in the dark. The cells were washed again three times with PBS, stained with 4′,6-diamidino-2-phenylindole (DAPI, Abcam, United States), and observed using a fluorescence microscope (LEICA, Germany).

##### 2.3.4.2 The Expression of Inflammatory Genes

RAW264.7 was seeded on bone cement at a cell density of 300,000 cells per well in 24-well plates. The cells were cultured for 1 days. Total RNA was extracted using an RNAprep Pure Cell/Bacteria Kit (TIANGEN, China), and reverse transcription was performed using a RevertAid First Strand cDNA Synthesis Kit (Thermo Fisher Scientific, United States). Then, a qRT-PCR test was performed using the real-time PCR system (Light Cycler 480, Roche, United States) with QuantiNova^TM^ SYBR Green PCR Master MIX (Qiagen, Germany). The target gene sequence primers, as shown in Supplementary Table S1, used GPDH as the house-keeping gene. The relative gene expression was calculated by Ct (2^−ΔΔCt^). All the test procedures followed the manufacturer’s instructions. Three samples were tested in each group.

##### 2.3.4.3 Enzyme-Linked Immunosorbent Assay

The RAW264.7 cells were incubated on the bone cements for 1 and 3 days. The cell supernatant was used to the measure cytokine concentration using an ELISA assay kit (R&D Systems, United States). The operation was done according to the manufacturer’s instructions and calibrated by standard curves. Three samples were tested in each group.

### 2.4 *In Vivo* Studies

#### 2.4.1 Bone Defect in Rats

Animal experiments were approved by the Ethics Committee of the University of Hong Kong-Shenzhen Hospital. A total of 24 3-month-old Sprague–Dawley rats were used and randomly divided into four groups: a CPC group, a 10% Mg-CPC group, and a 20% Mg-CPC group. Using 4% chloral hydrate anesthesia, the right leg was selected as the surgical site. After surgical site shaving and disinfection, a scalpel was used to expose the distal femoral, and a 2.5 mm-diameter, 4 mm-long hole was created by drilling the bone at the lateral epicondyle on the femur. The area was saline washed three times, and the CPC, 10% Mg-CPC, and 20% Mg-CPC samples were implanted in the defect areas of the rats in each group, respectively. The areas were then sutured layer by layer and disinfected, followed by routine feeding.

#### 2.4.2 Micro-CT Analysis

A CT scan was performed on the rats in each group at 4 and 8 weeks after the operations using a Micro-CT machine (Skyscan 1176, Bruker). The animals were then put into a respiratory anesthesia apparatus. After anesthesia, they were put into the slot of the micro-CT scanner, and a respiratory anesthesia mask was put on them. Then, each rat was placed on its side to fully expose the bone defect, the hatch was closed, and the scanning was started. After the scan, the animal was removed and the data were saved. Skyscan 1176 was used to select the scanning parameters: energy/intensity of 65 kVP, 385 A, a scanning time of 283 s, scanning accuracy of 18.04 μm, the bx2 mode, and a scanning angle of 0.5°, scanning 360° at once. NRecon software was used for 3D reconstruction, DataViewer was used for analysis, and CTan was used to draw VOI. We take ROI as a circle with a radius of 90 pixels and analyze a thickness of 0.722 mm. On this basis, the circle with a radius of 75 pixels was removed, and the remaining part is the range of our analysis. The analysis included the tissue volume (TV), the bone volume (BV), the relative bone volume or the bone volume fraction (BV/TV) and Bone mineral density (BMD).

#### 2.4.3 Histology

The rats were sacrificed, and their femurs were taken and fixed in 4% paraformaldehyde for 24 h at 4 and 8 weeks. The samples were decalcified using 10% EDTA, embedded in paraffin by an embedding machine (EG11504, Leica), and cut to 4 μm thick by a slicing machine (RM2235, Leica). The sections were stained with H&E (Solarbio) and Masson trichrome stain (Solarbio) and analyzed and photographed with a microscope panoramic scan.

### 2.5 Statistical Analysis

All the experiments had at least three independent replicates. The experimental chart was made using GraphPad Prism 7. All the results of each time point were presented as the mean ± standard deviation from three or more replicates. The statistical analysis was performed by Student’s *t*-test and one-way analysis of variance use SPSS 17. The *p* value <0.05 was considered statistically significant.

## 3 Results

### 3.1 The Material Characterization of Magnesium-Releasing Calcium Phosphate Bone Cement

#### 3.1.1 Surface Morphology and Magnesium Distribution

**FIGURE 1:**
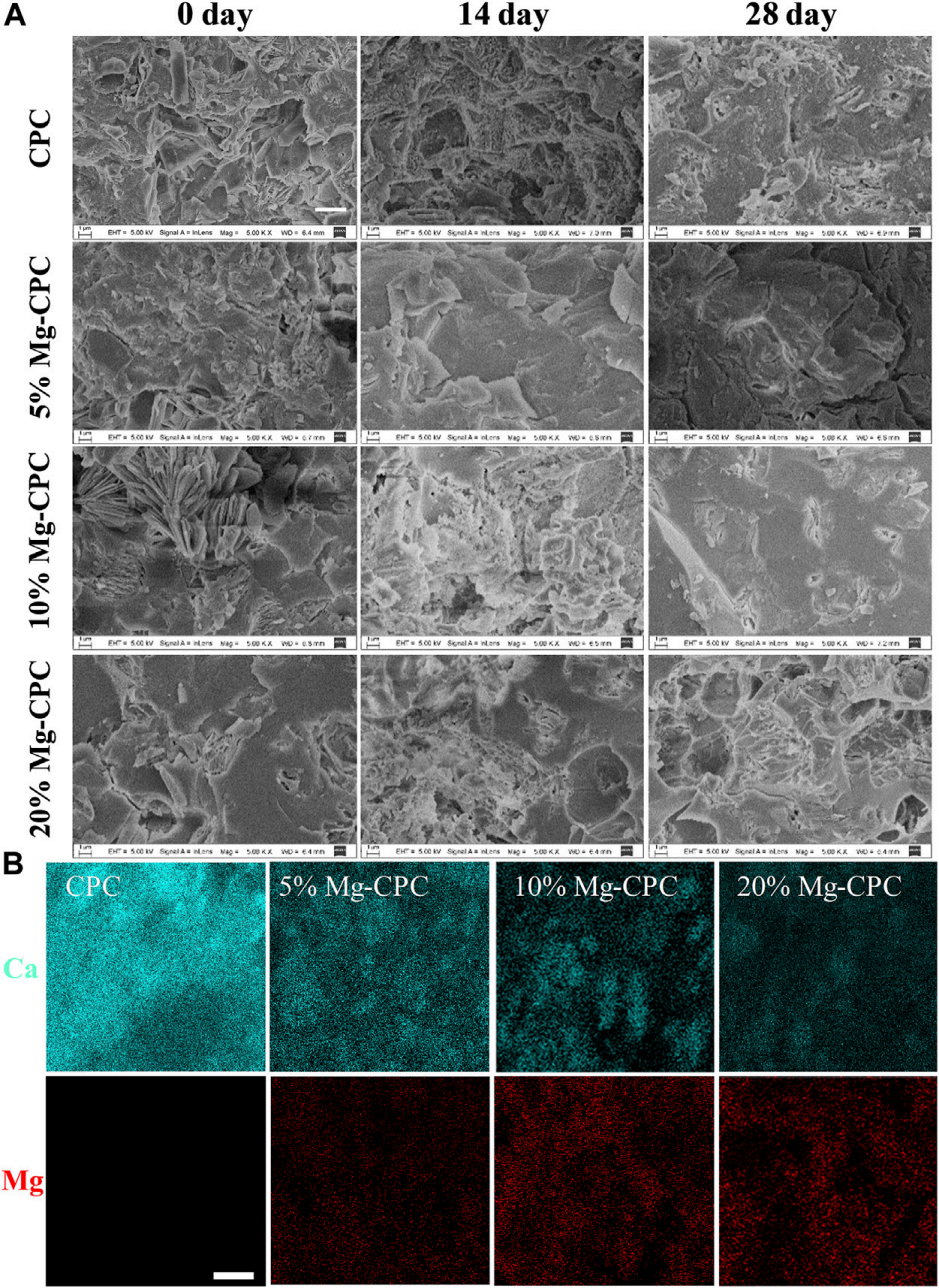
Surface morphology of CPC and Mg-CPCs samples by SEM. **(A)** The SEM images of CPC and Mg-CPCs samples after immersion in SBF (Scale bar = 3 μm). **(B)** EDS mapping of Ca (blue dots), Mg (red dots); EDS analysis elements’ composition on cement surfaces after immersion in SBF (Scale bar = 10 μm).

The SEM images of the CPC and Mg-CPC groups showed that after setting, similar irregular crystals could be observed (Figure 1A). As the immersion time increased, the irregular crystals gradually disappeared. After immersion in SBF for 28 days, the irregular crystals were almost disappeared. Porous structures could be observed in the Mg-CPC group, which was due to the release of magnesium (Figure 1A). The EDS showed that the calcium and the magnesium were uniformly distributed throughout the cement samples, and the content of Mg was increased by incorporating a higher ratio of magnesium phosphate dibasic trihydrate (Figure 1B).

#### 3.1.2 Crystal Structure

**FIGURE 2:**
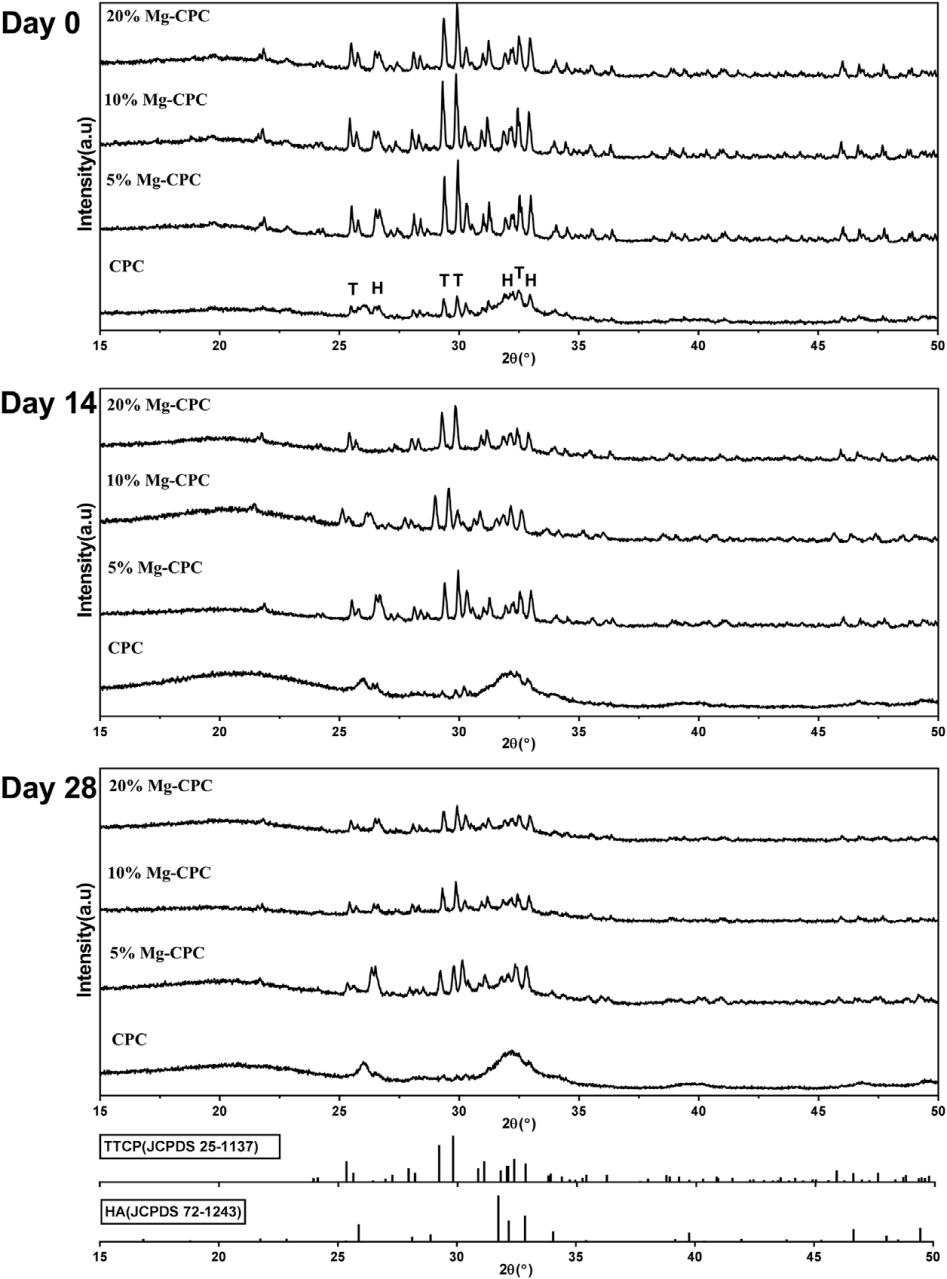
XRD patterns of cement types. After immersion in SBF 0, 14, and 28 days, all the cement groups were freeze-dried to remove moisture and then ground into a powder for tested. The results were compared with the standard card of HA (JCPDS 72-1243) and TTCP (JCPDS 25-1137) (H: HA, T: TTCP).

XRD patterns of CPC and Mg-CPC immersed in SBF were shown in Figure 2. Diffraction peaks of all raw materials could be observed at day 0, as well as diffraction peaks of HA, indicating that hydration reaction of TTCP is incomplete during cement solidification. As the immersion time increased, the intensities of diffraction peaks attributed to HA increased, while that of TTCP decreased, indicating that TTCP was hydrated to form HA gradually. At the day 28, the diffraction peak of TTCP disappeared and the broad diffraction peaks of HA was observed, indicating that TTCP in CPC group was completely hydrated and amorphous HA were formed. Diffraction peaks of TTCP and HA were detected in the Mg-CPC groups at day 28, indicating that the hydration rate of TTCP was slower in Mg-CPC groups.

#### 3.1.3 Mechanical Strength, Setting Time and *In Vitro* Ion Release

**FIGURE 3:**
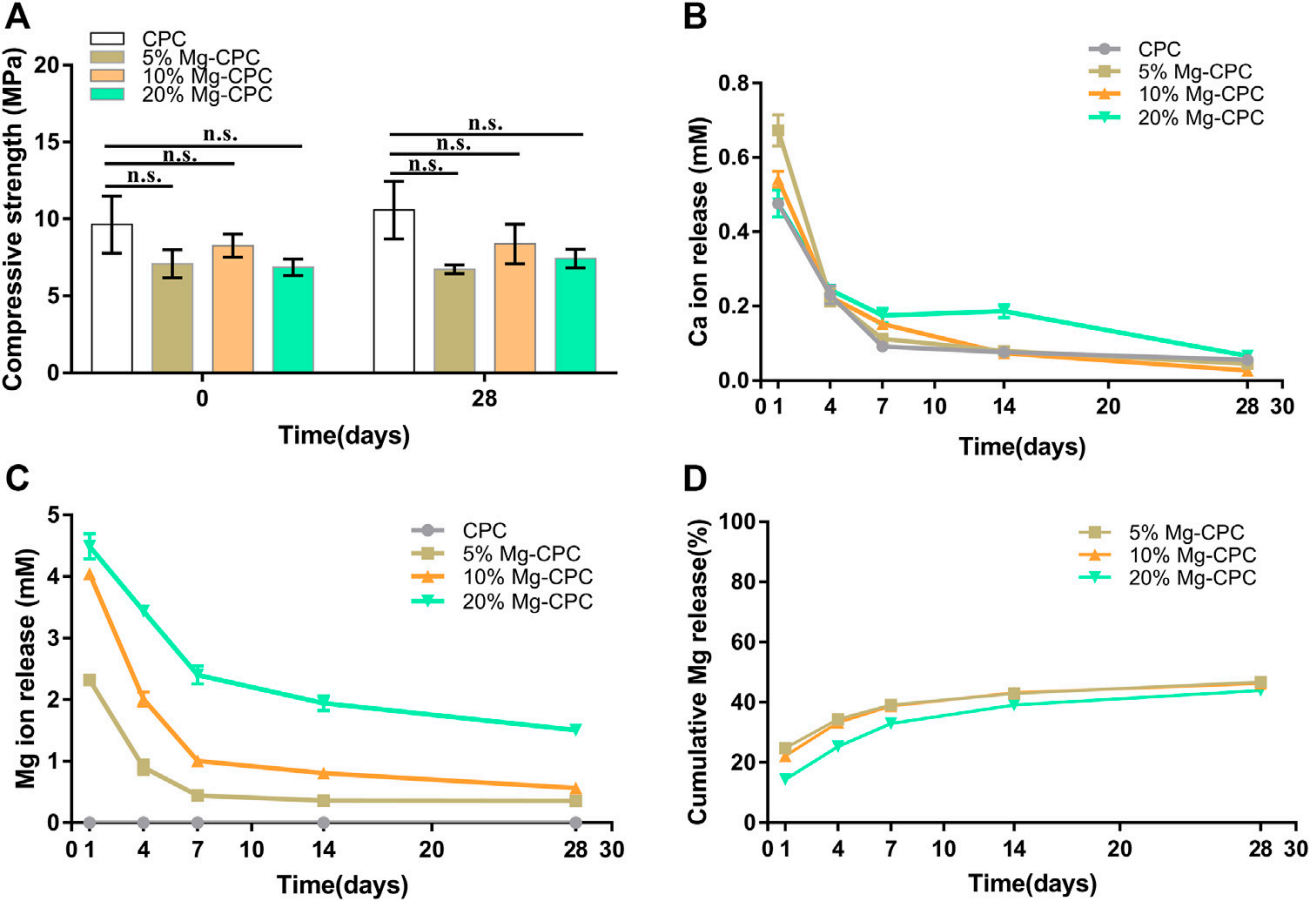
Material characterization of bone cement *in vitro*. **(A)** Compressive strength of CPC and Mg-CPCs samples after immersion in SBF at day 0 and 28 (n.s., no significant difference). **(B)** Ca released into PBS determined by ICP-MS. **(C)** Mg released into PBS determined by ICP-MS. **(D)** Cumulative release of Mg.

The compressive strength of the cement samples (Figure 3A) showed that, although the incorporation of Mg decreased the compressive modulus of CPC, their mechanical strengths were still close to cancellous bone. Furthermore, after immersion for 0 and 28 days, the compressive strengths of Mg-CPCs were almost unchanged, confirming the long-term mechanical stability of Mg-CPC. For comparison, MgO was also incorporated into CPC (MgO-CPC) as a substitution of magnesium phosphate dibasic trihydrate. After immersion in SBF, MgO-CPC quickly collapsed due to the fast degradation rate of MgO (Supplementary Figure S1).

The setting time of CPC and Mg-CPCs are shown in Table 2. The initial setting time of CPC and Mg-CPCs were about 10 min. The final setting time of Mg-CPCs decreased with the addition of DMPA compare with CPC. However, with the addition of DMPA increased, the setting time also increased.

**TABLE 2:** Compressive strength and setting time of cements.

Group	CPC	5% Mg-CPC	10% Mg-CPC	20% Mg-CPC
Compressive strength (MPa)				
Day 0	9.64 ± 3.21	7.08 ± 1.58	8.26 ± 1.31	6.85 ± 0.945
Day 28^a^	10.57 ± 3.25	6.73 ± 0.50	8.37 ± 2.22	7.42 ± 1.05
Setting time (min)				
Initial	10.86 ± 0.34	9.55 ± 0.34	9.89 ± 0.26	11.05 ± 0.25
Final	21.39 ± 0.51	15.5 ± 0.5	17.77 ± 0.25	19.38 ± 0.53

a The time of immersion in SBF.

The Ca ion concentration in the extract was shown in Figure 3B. An initial burst release was observed on day 1. After that, the release rate rapidly decreased. The ratio of Mg showed no effect on the release profiles of calcium.

The release profiles of Mg ion are shown in Figure 3C. Similar to Ca, initial burst releases on day 1 were observed in all the groups. After that, the release rates of Mg rapidly decreased until day 7, when near zero-order releases were achieved.

#### 3.1.4 Effects of Magnesium-Releasing Calcium Phosphate Bone Cement on Human Bone Marrow Mesenchymal Stem Cells

**FIGURE 4:**
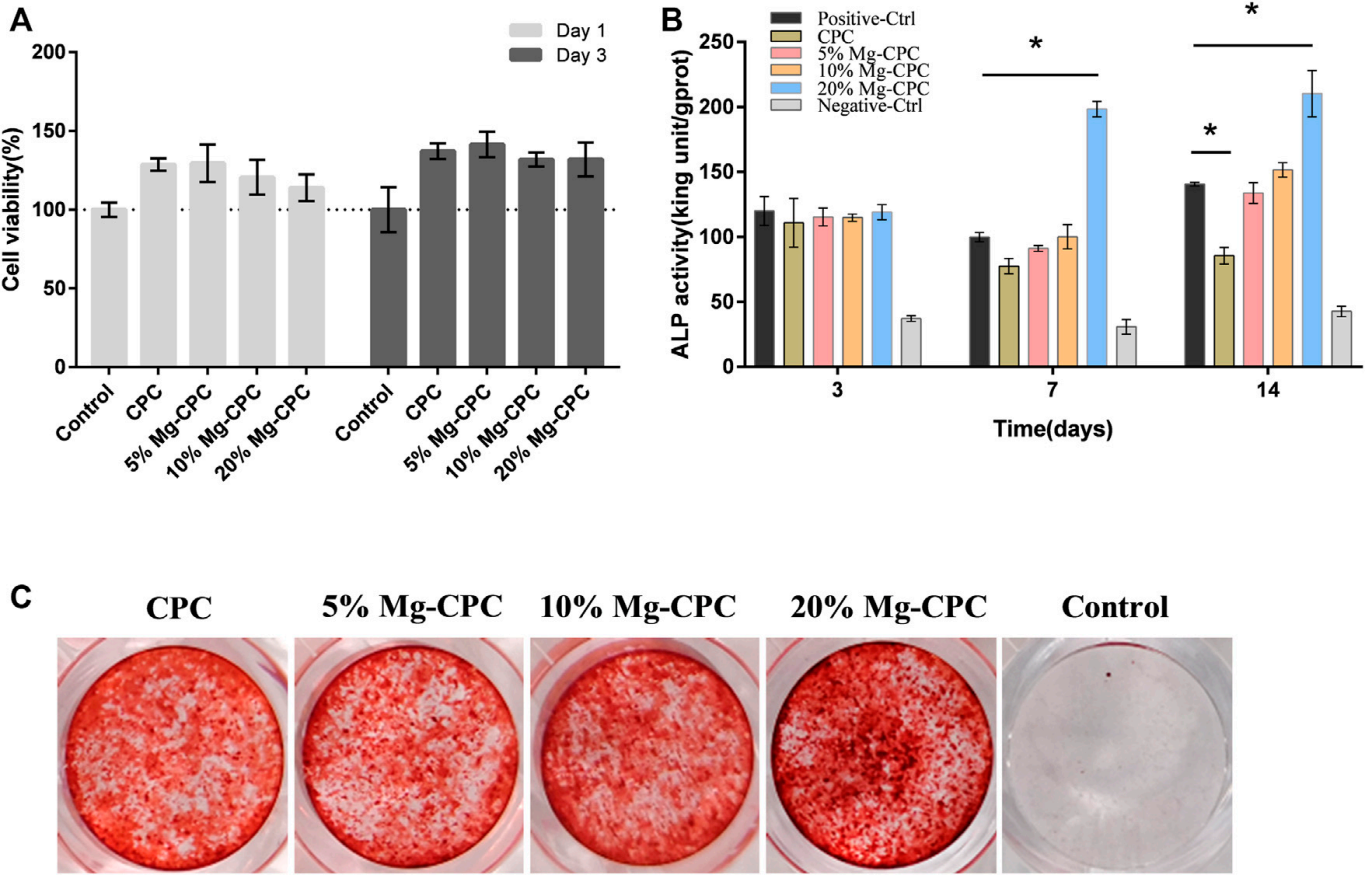
Effect of cement on hBMMSCs. **(A)** Cell viability assay of CPC and Mg-CPCs. **(B)** ALP activity assay of CPC and Mg-CPC. **(C)** Alizarin red staining of CPC and Mg-CPCs (*p < 0.05).

To verify the effect of Mg-CPC on hBMMSCs, we used bone cement extract to test cytotoxicity, ALP activity, and *in vitro* mineralization. As shown in the figure, we tested the cell activity on day 1 and day 3 under the extract culture conditions and found that the cell viabilities of all the cement groups were higher than that of the blank control group, and none of the cement groups showed any toxicity to hBMMSCs. Indicating that all the cement samples had good biocompatibility (Figure 4A).

In the ALP activity test, we detected ALP activity on day 3, day 7, and day 14 and found that on day 3, no difference was observed between the cement group and the control group. The ALP activity of the 20% Mg-CPC group significantly increased on day 7 and day 14 (Figure 4B).

The mineralization effects of the Mg-CPCs were evaluated using alizarin red staining, which stained the calcium nodules in ECM into a red color. The results showed that the 20% Mg-CPC group exhibited more calcium nodules compared with the control group and the other Mg-CPC groups. The ALP test and alizarin red staining demonstrated that 20% Mg-CPC had the best osteogenic effects.

#### 3.1.5 Animal Study

**FIGURE 5:**
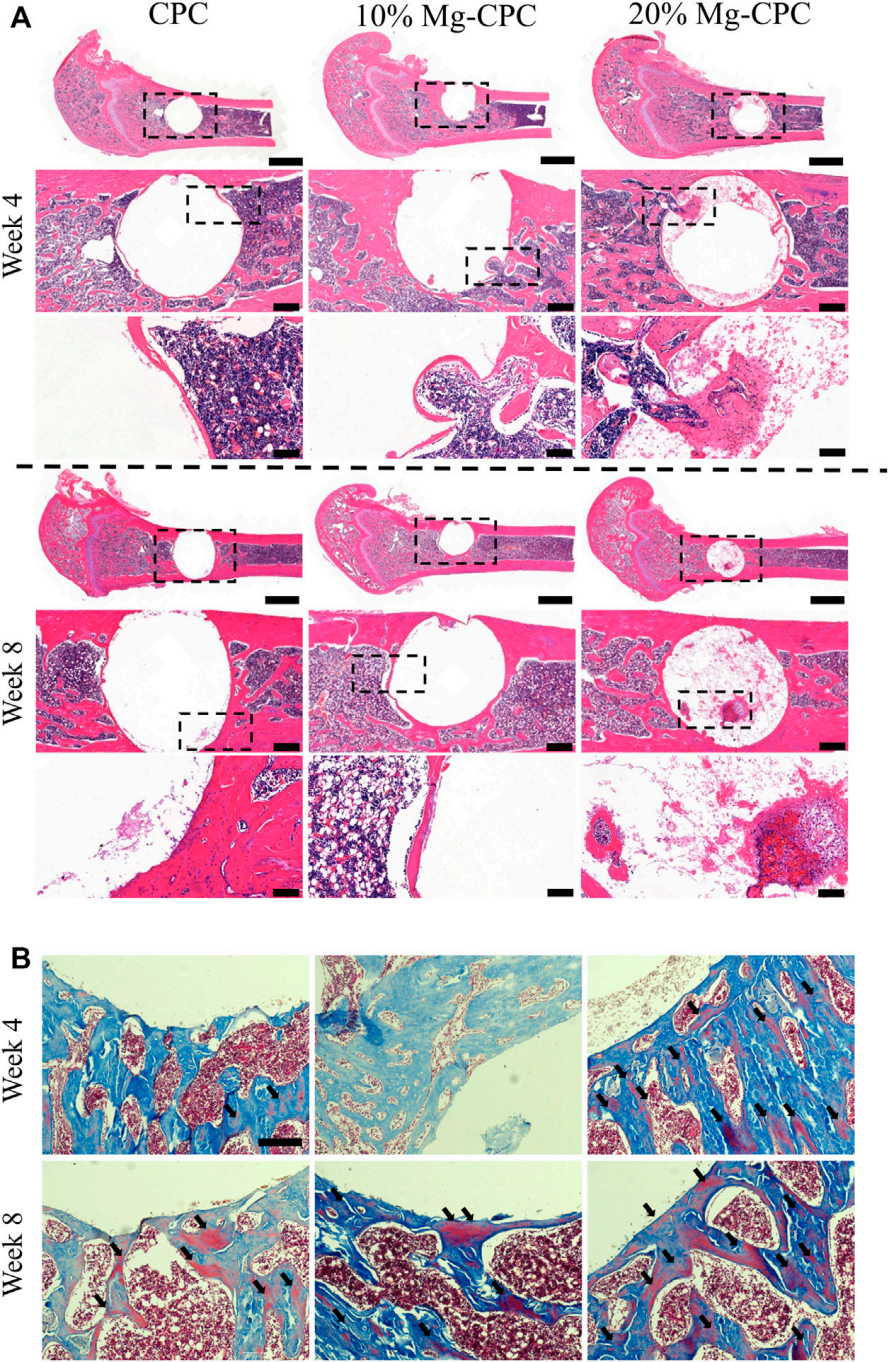
Histological staining after surgery. **(A)** H&E staining (scale bar = 2,000, 500, 100 μm); **(B)** Masson trichrome stain, red color represents mature bone (back arrow). Scale bar: 200 μm.

To investigate the osteogenic effect of magnesium release cement *in vivo*, we used a rat model of a critical bone defect of the femur (Figure 5A). Histological staining was performed at 4 and 8 weeks after bone cement implantation. Decalcification was performed without the implant being removed. The H&E staining results showed that a small amount of bone tissue could be observed in 20% Mg-CPC at week 4, but not in the control group. Interestingly, a large amount of bone tissue was observed in the 20% Mg-CPC at week 8 (Figure 5A), suggesting new bone had thoroughly grown into the 20% Mg-CPC. To observe bone maturation, mason trichrome stain was used. Mature bone (red color) could be observed in the 20% Mg-CPC at week 4, which was not observed in the CPC group. More red color appeared in the 20% Mg-CPC group at week 8, suggesting more mature bone in the 20% Mg-CPC group (Figure 5B).

**FIGURE 6:**
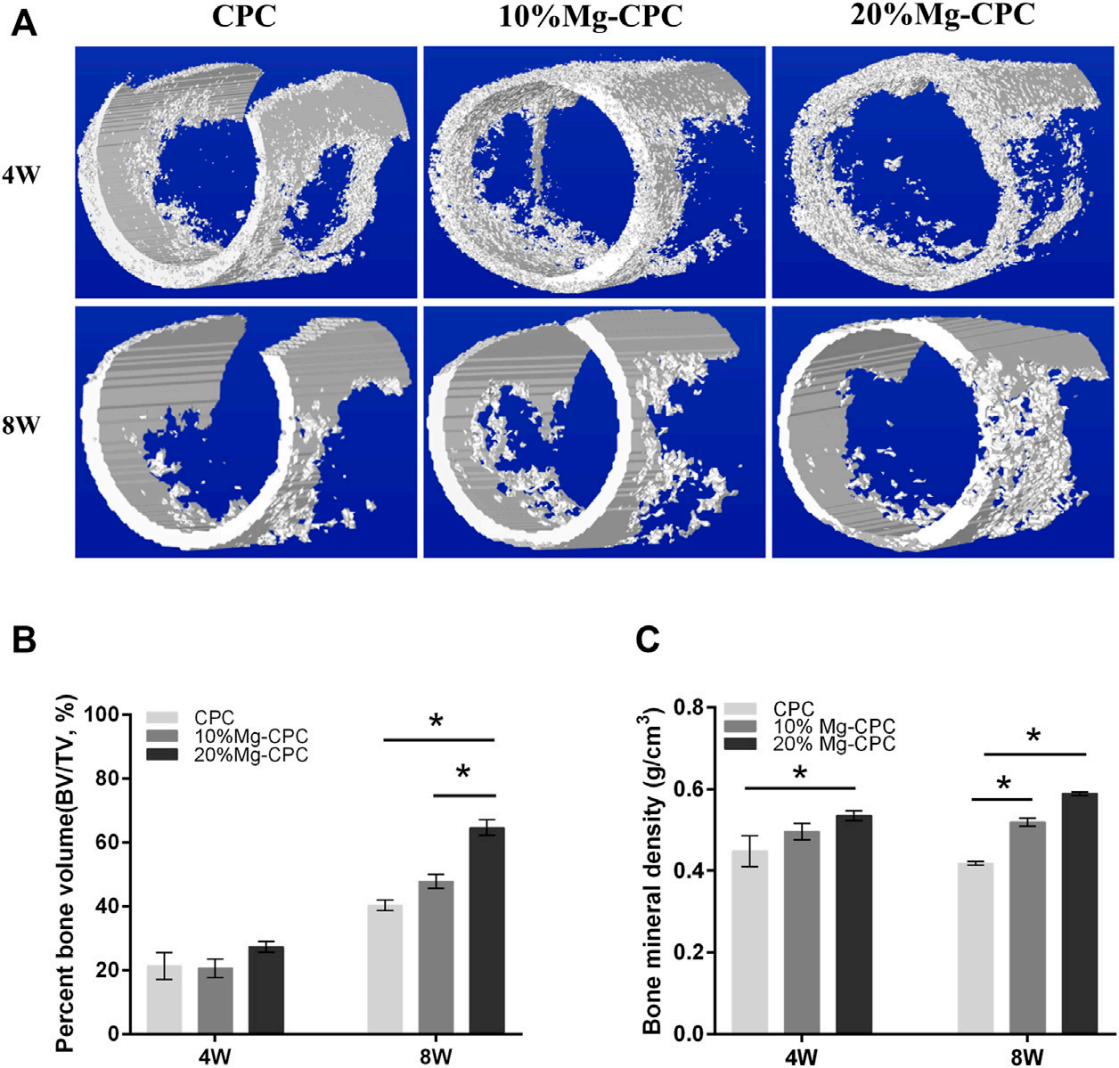
Micro-CT evaluation after surgery. **(A)** 3D reconstruction models of new bone formation at the defect sites at different time points; **(B)** The Percent bone volume (BV/TV) of newly formed bone tissue at 4 and 8 weeks after surgery; **(C)** The bone mineral density values of different cement groups at 4 and 8 weeks after surgery (*p < 0.05).

Micro-CT evaluations were performed at 4 and 8 weeks after surgery. To visually observe the new bone formation, we performed a three-dimensional reconstruction of the bone defect site (Figure 6A). No significant difference was found in the bone volume of the new bone formation between the CPC and the Mg-CPCs at week 4. The bone volume of newly formed bone was significantly different in the 20% Mg-CPC group compared to the CPC and 10% Mg-CPC group at week 8 (Figure 6B). By calculating the BMD, we found that there was a significant difference between 20% Mg-CPC and CPC at week 4. However, at the week 8, the BMD was significantly different in Mg-CPC groups compared to the CPC group, with 20% Mg-CPC groups having the highest BMD (Figure 6C).

#### 3.1.6 Effects of Magnesium-Releasing Calcium Phosphate Bone Cement on RAW 264.7

**FIGURE 7:**
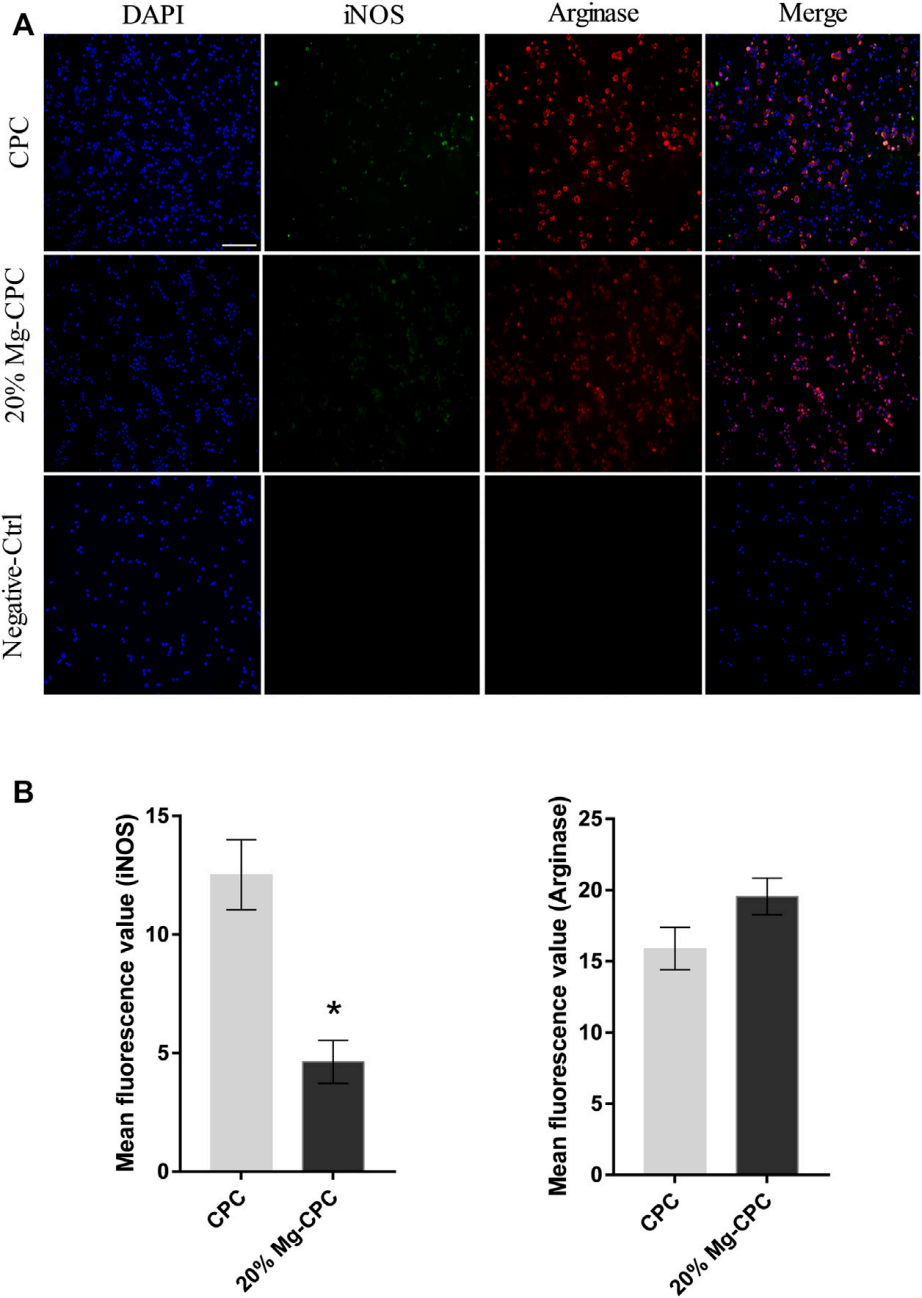
Immunofluorescence staining of iNOS and Arginase in RAW 264.7. **(A)** Fluorescence images of RAW 264.7 cultured on cement for 24 h; blue dot: DAPI; green dot: iNOS; red dot: arginase (scale bar = 200 μm); **(B)** mean fluorescence intensity calculation by software ImageJ (*p < 0.05).

Immunofluorescence staining analysis of the polarization of the macrophages (Figure 7A) in the negative-control group did not show signs of iNOS and arginase. In all the cement groups, iNOS and arginase obtained different degrees of signal expression; the iNOS signal was weaker compared with the arginase signal, indicating that CPC could simultaneously promote the polarization of RAW 264.7 toward the M1 and M2 types, while the effect of promoting the polarization of the M2 type was stronger.

By comparing the mean fluorescence intensity of the two kinds of cements, we found no significant difference in the M2 markers between them, but CPC expressed more M1 markers (Figure 7B).

**FIGURE 8:**
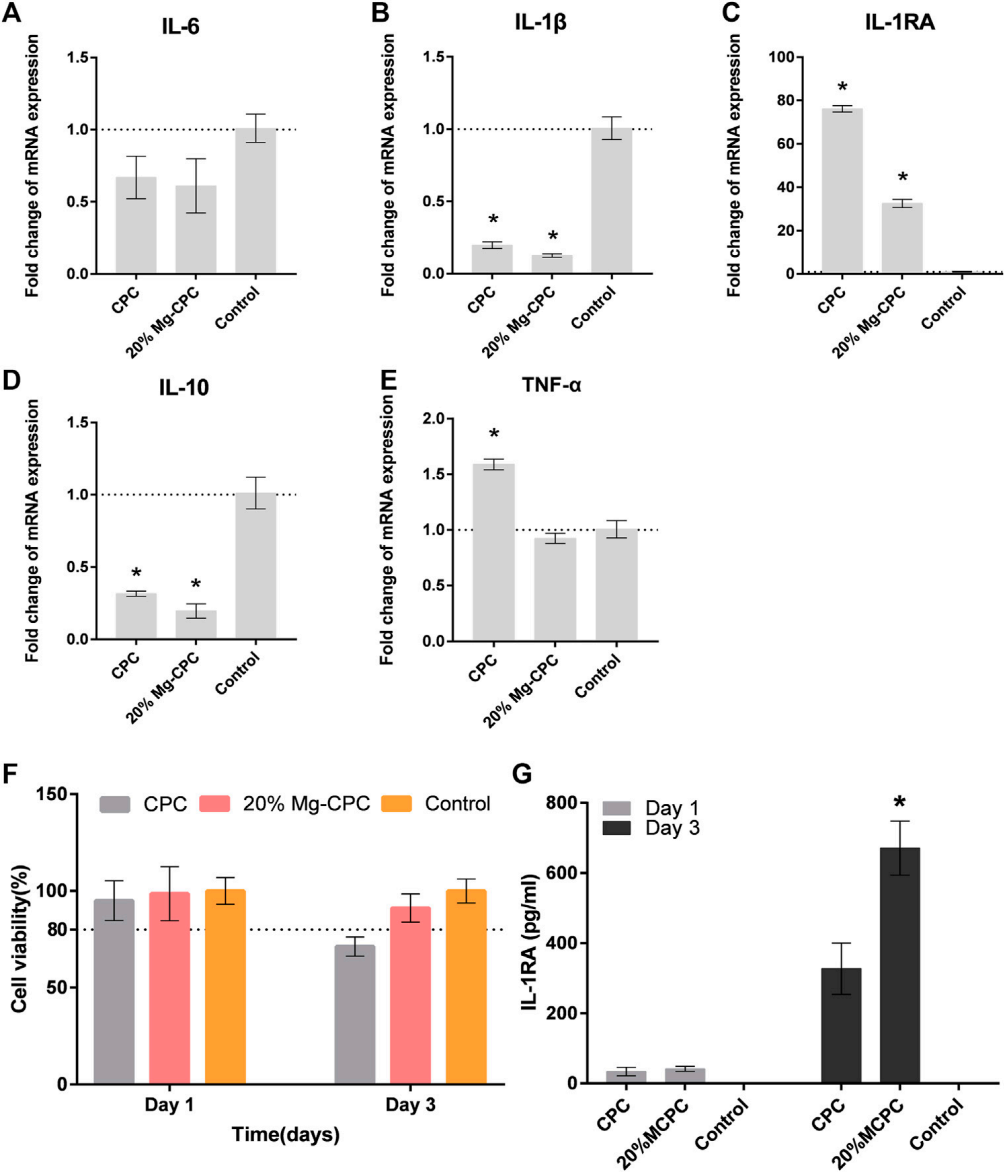
The effect of CPC and Mg-CPC on RAW264.7. **(A–E)** Gene expression of IL-6, IL-1β, IL-1Ra, IL-10, and TNF-α after culture on cement, detected by real-time PCR assay. **(F)** Cell viability assays. **(G)** The concentration of IL-1RA in the supernatant of RAW264.7 using ELISA (*p < 0.05).

To verify magnesium-releasing CPC of macrophage RAW264.7 cell toxicity in mice, we used the bone cement extract for the cytotoxicity test (Figure 8F). Under the condition of extract culture, we tested the cytotoxicity at 1 and 2 days. On the first day all the cement groups showed no toxicity compare with the control group, however, CPC cell activity was lower than 80% in the control group, indicating cell toxicity at day 2.

To measure the gene expression of pro-inflammatory cytokines and anti-inflammatory cytokines, a q-PCR test was used. The expression of TNF-α (Figure 8E), IL-6 (Figure 8A), and Il-1β (Figure 8B) of the pro-inflammatory cytokine were all lower in MG-CPC than in the blank control group. The 20% Mg-CPC inhibited the gene expression of pro-inflammatory cytokine. In terms of the expression of anti-inflammatory cytokine genes, both CPC and Mg-CPC promoted the expression of IL-1RA (Figure 8C), but showed no effect on the expression of the IL-10 (Figure 8D) gene. A further ELISA assay showed that the concentration of IL-1RA in the 20% Mg-CPC had significantly increased at day 3 (Figure 8G) compared to the CPC group.

## 4 Discussion

Evaluations of bioactive materials are suitable for clinical inquiry and are important in determining biological characteristics. CPC has good biological characteristics. For example it is similar to the composition of bone in terms of its hydration properties. Moreover, its absorption rate is consistent with the speed of new bone formation, it does not affect the bone healing process, it is easy to shape, and it has no obvious side effects. Its potential clinical applications is strong compared to other bioactive materials (Won et al., 2010). Unfortunately, the effect of CPC alone on osteogenesis is limited, which may cause bone healing delay or even implant failure. Many researchers have improved CPC; however, few studies have been done on the mechanisms of its limited osteogenic effects. Immune regulation plays an important role in osteogenesis, and the limited osteogenic qualities of CPC may be related to immune regulation. Magnesium has excellent bone-promoting and immune regulation abilities (Hu et al., 2018; Nabiyouni et al., 2018). Therefore, magnesium was introduced into CPC in this study to examine the causes and mechanisms of its limited bone-promoting ability.

CPC is highly biocompatible with almost no toxicity, while magnesium, as one of the elements of human body, has no cytotoxicity in the appropriate concentration range and has demonstrated good biological safety (Feyerabend et al., 2010). This study also found no cytotoxicity in the treatment of hBMMSCs (Figure 4A). Many articles have reported the beneficial effect of magnesium ions on bone formation, and they can regulate the osteogenic differentiation of hBMMSCs in various ways to promote bone healing. Studies have found that magnesium ions can significantly improve the activity of alkaline phosphatase and the expression of osteogenic-related genes, promote the osteogenic differentiation of mouse MC3T3-e1 pre-osteoblasts, and promote the mineralization of the extracellular matrix (Wong et al., 2013). Magnesium ions can enter the periosteum and induce neurons to produce a calcitonin gene-related polypeptid-α (CGRP) and stimulate periosteum-derived stem cells (PDSCs) to undergo osteogenic differentiation to promote bone healing (Zhang et al., 2016). The 20% Mg-CPC synthesized in this study could significantly improve the activity of ALP and promote the mineralization of ECM (Figure 4C), while the ALP activity of CPC significantly decreased on day 14 (Figure 4B), indicating that CPC had an inhibitory effect on the osteogenic differentiation of hBMMSCs. In addition to the decreased ALP activity, other mechanisms of inhibiting hBMMSCs osteogenic differentiation need to be demonstrated in further studies. The animal study showed consistent results the *in vitro* study, and the 20% Mg-CPC showed better osteoconduction (Figure 5) and osteogenesis (Figure 6).

Immunomodulatory function is believed to play a key role in the process of bone healing (Takayanagi, 2007). The bone healing delay caused by CPC is also related to the effect of CPC on immune regulation. When the macrophage RAW264.7 was treated with extracts of CPC and Mg-CPC, the Mg-CPC group had no cytotoxicity, but the CPC group showed cytotoxicity (Figure 8F), indicating that CPC has a negative effect on immune cells that was ameliorated by magnesium ions. Macrophage polarization is one of the manifestations of immune regulation. M1-type macrophages promote inflammation, and M2-type macrophages promote wound healing. The immunofluorescence staining in this study showed that the two phenotypes of M1-type and M2-type macrophages coexisted, but there were more M1-type macrophages in CPC group than in Mg-CPC group (Figure 7). The NF-κB signaling pathway is closely related to inflammatory and immune responses, and the activation of the NF-κB signaling pathway promotes the expression of pro-inflammatory cytokines, which further activates the NF-κB signaling pathway. Sustained activation of the NF-κB signaling pathway leads to long-term inflammatory responses that lead to cellular damage and inhibit osteogenic differentiation (Lin et al., 2017). In this study, the anti-inflammatory cytokine IL-1RA gene was significantly up-regulated in Mg-CPC-treated macrophages; a further ELISA assay showed that the concentration of IL-1RA had significantly increased. The anti-inflammatory cytokine IL-1RA effectively blocked the binding of IL-1 to its membrane-bound receptor IL-1RI (Gabay et al., 2010), thereby inhibiting the NF-κB pathway activation. In macrophages treated with CPC, inhibiting inflammatory cytokines IL-1RA have also been raised, but the pro-inflammatory cytokines TNF-α gene increases, and the TNF-α, IL-6, and IL-1β genes in macrophages treated with Mg-CPC groups were down-regulated (Figures 7A–E). TNF-α can continuously activate the NF-κB pathway (Wang et al., 2017), produce inflammatory cytokines, and inhibit bone formation by blocking the Wnt signaling pathway (Vincent et al., 2009). The release concentrations of calcium ion were similar between the CPC group and the Mg-CPC groups. In summary, CPC is cytotoxic to macrophage RAW264.7 *in vitro* and promotes the secretion of TNF-α by macrophage RAW264.7 to activate the NF-κB pathway, promote an inflammatory response, and inhibit osteogenic differentiation that leads to a bone healing delay (Ye et al., 2016; Yu et al., 2020). The introduction of magnesium can ameliorate these adverse effects and inhibit TNF-α production, thereby reducing the release of pro-inflammatory cytokines (Weglicki et al., 1992).

Magnesium is one of indispensable elements in the human body’s life activities. A large number of studies have found that the mechanical properties of magnesium alloys can promote bone growth *in vivo* along with biological activity to accelerate fracture healing. However, magnesium alloys will gradually and eventually degrade (Witte, 2010), and the corrosion rate of magnesium alloys is too fast; it cannot provide mechanical support for a long time, and its corrosive byproducts are harmful to the local microenvironment (Wong et al., 2010), limiting its clinical application. Therefore, this study introduced magnesium into calcium phosphate cement to explore the potential of this combined material.

The water-resistance and collapsibility of bone cement is an important factor for its clinical application. If rapid collapse occurs after bone cement is implanted, it cannot provide a support role, and it also produces a large number of vesicles containing inflammatory exudates. Moreover, it increases the infiltration of inflammatory cells, aggravates the inflammatory response, and may result in embolism formation in the blood vessels, which are serious consequences (Miyamoto et al., 1999). In our previous studies, we have accelerated the solidification of CPC by optimizing concentration of citric acid in liquid phase, which functioned as a chelation agent for calcium ions (Kuang et al., 2012). In this study, citric acid in the liquid phase can chelate calcium ions and magnesium ions to form complex, accelerate cement solidification, and resist water and collapse. PVP in the liquid phase were increase the viscosity of water agent, and stability of the system. PVP could also improve the thixotropy after mixing, which would improve the injectability and facilitate to shaping of bone cement in the clinical requirements. The water resistance experiment results show that the MgO group quickly collapsed in a PBS solution, but the magnesium and hydrogen phosphate groups still maintained complete form after immersion in PBS for 3 months (Supplementary Figure S1). It showed that the addition of MgO had a great influence on the bone cement system, and its water-resistant ability was directly lost. It suggested that hydration products of MgO disrupt the crosslinking network of the CPC system. However, magnesium hydrogen phosphate can be well integrated into a CPC system so that it can maintain good water resistance and collapse-resistant ability.

Mg-CPC offers long-term stable magnesium release and mechanical strength. Even as a non-weight-bearing bone repair material, bone cement can maintain a certain mechanical strength for a long time, which is very important for clinical applications (Yetkinler et al., 2001). In this study, the mechanical strength of the Mg-CPC decreased compared with bone cement (Figure 3A), indicating that the introduction of magnesium had an adverse effect on the original mechanical strength. This adverse effect occurs after the Mg-CPC has solidified. After immersion in SBF and using SEM to observe the surfaces of the bone cement groups (Figure 1A), the crystals gradually disappeared as the hydration reaction progressed, and the HA in the cement gradually increased, as determined by XRD (Figure 2). With an increase of HA, the mechanical strength should be enhanced (Lacout et al., 1996). However, no increase in mechanical strength was observed in our results, which may be due to the release of the magnesium ions. In the Mg-CPC groups, a large number of magnesium ions appeared to be releases in the first 3 days, and the subsequent release became stable (Figure 3C). The release of magnesium accounted for more than 40% of the added weight at day 28 according to theoretical calculations, while the release of calcium in all the groups was negligible. Magnesium release reduces mechanical properties, while HA formation increases mechanical properties. To sum up, our results showed that, the sustained release of magnesium and long-term mechanically stable can be achieved at the same time by synergistic effects of DMPA and citric acid. Based on the chemical properties of magnesium hydrogen phosphate dissolved in dilute acid, DMPA dissolves in citric acid and the released magnesium ions are chelated by citric acid and citric acid also can chelate the calcium ions of TTCP in the early hydration reaction (Yu et al., 2019). As the chelation reaction progresses, chelate complex and the hydration products to form the cross-linking network, slow down the TTCP hydrolysis (Shi et al., 2019; Zhong et al., 2020), that’s why we can still observe the diffraction peak of TTCP in the XRD pattern on day 28 (Figure 2). Although will slow down the formation of HA, but the cross-linking network can improve the water resistance ability of Mg-CPC, so that it is not to collapse in water (Supplementary Figure S1), and maintain long-term mechanical stability (Figure 3A). After setting, the PH value increases, the solubility of DMPA decreases and slowly hydrolyzes in water, therefore, the magnesium ions sustained release have achieved.

In conclusion, the poor ability of CPC to promote bone healing is mainly manifest in two aspects: stem cell osteogenesis and immune osteogenesis. *In vitro* study, CPC had no cytotoxic effects on hBMMSCs, but inhibited ALP activity at day 14, was cytotoxic to macrophages, and promoted the secretion of the pro-inflammatory cytokine TNF-α by macrophage RAW 264.7. Mg-CPC can enhance the activity of ALP, the mineralization ability on hBMMSCs, suppress the M1 polarization of macrophage and the expression of anti-inflammatory cytokine IL-1RA on macrophage RAW 264.7. In the animal study, magnesium ions were shown to improve the osteoconduction ability of CPC. In addition, the Mg-CPC synthesized in this study had anti-collapsibility, long-term stable mechanical properties and showed no significant difference in compressive strength from CPC; it also showed a magnesium sustained-release capability. These factors suggest that Mg-CPC should be a good substitute material for bone grafts in clinical use.

## Data Availability

The raw data supporting the conclusion of this article will be made available by the authors, without undue reservation.
