# Corrigendum: The Development of a Magnesium-Releasing and Long-Term Mechanically Stable Calcium Phosphate Bone Cement Possessing Osteogenic and Immunomodulation Effects for Promoting Bone Fracture Regeneration

**DOI:** 10.3389/fbioe.2022.887252

**Published:** 2022-04-04

**Authors:** Jun Wu, Feihong Liu, Zejin Wang, Yuan Liu, Xiaoli Zhao, Christian Fang, Frankie Leung, Kelvin W. K. Yeung, Tak Man Wong

**Affiliations:** ^1^ Shenzhen Key Laboratory for Innovative Technology in Orthopaedic Trauma, The University of Hong Kong-Shenzhen Hospital, Shenzhen, China; ^2^ Department of Orthopaedics and Traumatology, The University of Hong Kong, Hong Kong, China; ^3^ Research Center for Human Tissues and Organs Degeneration, Institute of Biomedicine and Biotechnology, Shenzhen Institutes of Advanced Technology, Chinese Academy of Sciences, Shenzhen, China

**Keywords:** calcium phosphate cement, immunomodulation, osteogenic, bone regeneration, anti-collapsibility

In the original article, there was a mistake in Figure 1A as published. A mismatched image was uploaded to SEM image of CPC group at day 0. The corrected Figure 1 appears below.

**FIGURE 1:**
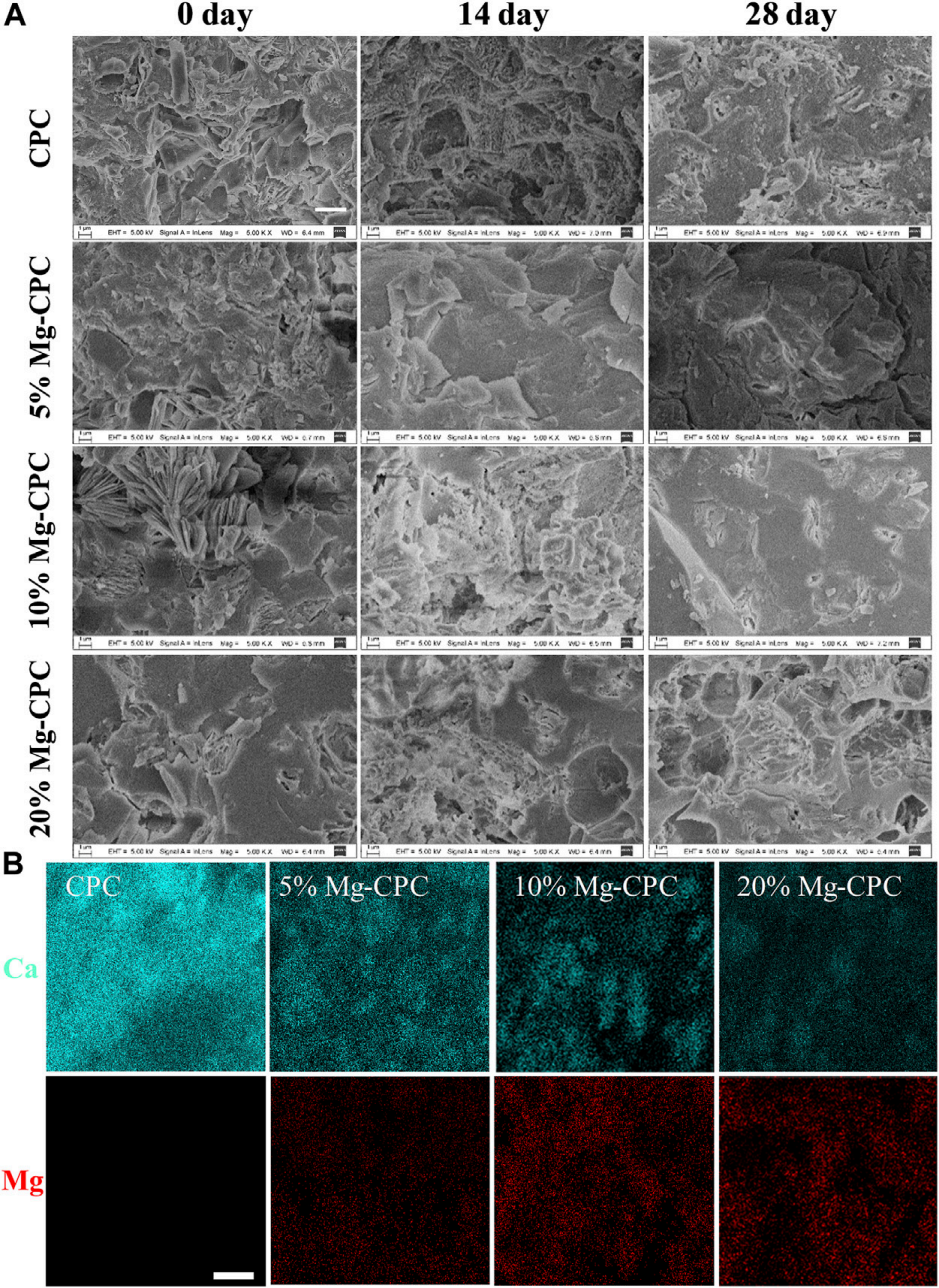
Surface morphology of CPC and Mg-CPCs samples by SEM. **(A)** The SEM images of CPC and Mg-CPCs samples after immersion in SBF (Scale bar = 3 μm). **(B)** EDS mapping of Ca (blue dots), Mg (red dots); EDS analysis elements’ composition on cement surfaces after immersion in SBF (Scale bar = 10 μm).

The authors apologize for this error and state that this does not change the scientific conclusions of the article in any way. The original article has been updated.

