# Supplementary material for: The Development of a Magnesium-Releasing and Long-Term Mechanically Stable Calcium Phosphate Bone Cement Possessing Osteogenic and Immunomodulation Effects for Promoting Bone Fracture Regeneration
Source: Front Bioeng Biotechnol. 2022 Jan 11;9:803723. doi: 10.3389/fbioe.2021.803723 (PMC8786730; doi:10.3389/fbioe.2021.803723)
Supplement: Supplementary file 1 [file DataSheet1.PDF]

Supplementary Tables 1. Primer sequences

Target gene	Direction	Sequence
GAPDH (mouse)	Forward	5'-TGGCCTTCCGTGTTCCTAC-3'
	Reverse	5'-GAGTTGCTGTTGAAGTCGCA-3'
IL-6	Forward	5'-ATAGTCCTTCCTACCCCAATTTCC-3'
	Reverse	5'-GATGAATTGGATGGTCTTGGTCC-3'
IL-1β	Forward	5'-TGGAGAGTGTGGATCCCAAG-3'
	Reverse	5'-GGTGCTGATGTACCAGTTGG-3'
IL-10	Forward	5'-GAGAAGCATGGCCCAGAAATC-3'
	Reverse	5'-GAGAAATCGATGACAGCGCC-3'
IL-1Ra	Forward	5'-CTCCAGCTGGAGGAAGTTAAC-3'
	Reverse	5'-CTGACTCAAAGCTGGTGGTG-3'
TNF-α	Forward	5'-CTGAACTTCGGGGTGATCGG-3'
	Reverse	5'-GGCTTGTCACTCGAATTTTGAGA-3'

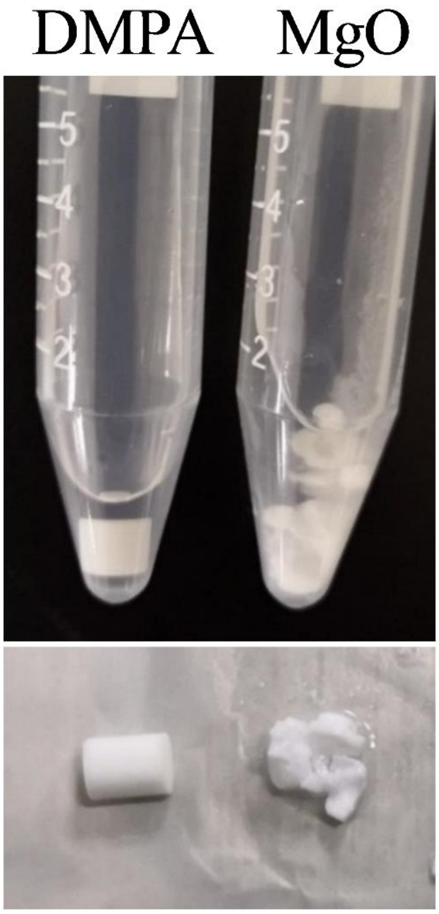

\

Supplementary Figure 1. Mg-CPC and MgO-CPC immersion in PBS (Left: collapse resistant Mg-CPC, Right: normal Mg-CPC).
